# Navigating life's challenges: A randomized 6‐week online intervention study to enhance resilience in working‐age adults

**DOI:** 10.1111/aphw.70023

**Published:** 2025-03-30

**Authors:** Manuel Leitner, Andreas Fink, Viktoria Fruhwirth, Stefanie Hechenberger, Christian Enzinger, Daniela Pinter

**Affiliations:** ^1^ Department of Neurology Medical University of Graz Graz Austria; ^2^ Department of Psychology, Section of Biological Psychology University of Graz Graz Austria; ^3^ Department of Neurology, Research Unit for Neuronal Plasticity and Repair Medical University of Graz Graz Austria

**Keywords:** intervention, online, resilience, stress, working‐age individuals, worries

## Abstract

Psychological resilience describes a trainable capacity that allows us to cope with adversity and adapt to stressful life events. This study aimed to investigate the efficacy of a 6‐week online resilience intervention in students and working‐age individuals.

We randomly assigned 174 participants aged between 18 and 54 years (74.1% female) to either an intervention (*n* = 86, *M*_age_ = 24.83, *SD*_age_ = 5.93) or a wait‐list control group (*n* = 88, *M*_age_ = 23.30, *SD*_age_ = 4.49). The intervention group received a 6‐week flexible, asynchronous online resilience training consisting of 12 short videos. We assessed resilience, perceived stress, and worries in both groups.

Mixed‐ANCOVA results indicated that participants assigned to the intervention group significantly increased their resilience scores (*M*_*diff*_ = 3.65, *p* < .001) and reduced negative emotions such as perceived stress (*M*_*diff*_ = 4.18, *p* < .001) and worries (*M*_*diff*_ = 5.09, *p* < .001). We observed no significant changes in the wait‐list control group.

The intervention group's ability to enhance resilience through watching two short videos per week supports the idea that resilience is trainable. Online resilience interventions represent a promising opportunity to acquire novel coping strategies in order to strengthen resilience and manage life's upcoming challenges.

## INTRODUCTION

Especially during demanding times in which individuals are confronted with serious risks such as armed conflicts, natural disasters, or pandemics, research on enhancing resilience became imperative to counteract rising anxiety and depression rates within our society (Brouzos et al., 2021; Kimhi et al., 2023; Salari et al., 2020; Yap et al., 2023). Notably, the Covid‐19 pandemic is an example where resilience‐enhancing interventions were shown to mitigate the global mental health burden (Yap et al., 2023). However, resilience can also help to cope with common day‐to‐day challenges such as stress or worries (Diehl et al., 2012). Although stress is generally perceived as negative, research from recent years shows that stressors can also have a resilience‐strengthening role if individuals have access to the required resources and perceive themselves as capable of managing them (Crane & Searle, 2016; Crane et al., 2018). Hence, training programs that target positive attitudes, cognitive reframing, and resource management may help mitigate the negative effects of adverse life circumstances and foster resilience in everyday life (Joyce et al., 2018).

Resilience describes the capacity to thrive in the face of adversity (Connor & Davidson, 2003) and should not be viewed as a static construct but rather as a dynamic and adaptive process that develops through ongoing and bidirectional interactions between individual, social, cultural, and systemic factors (Hill et al., 2018; Liu et al., 2017). According to Rampe (2010), resilience is based on seven pillars, including optimism, acceptance, and solution orientation, leaving the victim role, taking responsibility, network orientation, and planning for the future. Optimism describes a person's ability to maintain a positive outlook and to be aware that situations can change for the better; acceptance refers to acknowledging situations, even if they cannot be changed; solution orientation focuses on addressing challenges to achieve personal goals; leaving the victim role describes the ability to take life into your own hands; taking responsibility refers to feeling responsible for your own decisions; network orientation focuses on building a supportive network and seeking help if needed; and planning for the future focuses on goal setting and making plans even in times of uncertainty (Rampe, 2010).

Studies consistently found a negative association between resilience and various psychological domains such as perceived stress (Smith et al., 2008) and worries (Gordon et al., 2022; Tamarit et al., 2022), both of which are also associated with psychiatric disorders like major depression (Hammen, 2005; Yılmaz, 2015). In the latter, not only the treatment of symptoms but also the development of resilience skills can play a crucial role in preventing the long‐term recurrence of symptoms (Waugh & Koster, 2015). Hence, resilience training programs can be used as a preventive approach to build psychological strength, improve well‐being (Ang, Chew, et al., 2022), and reduce negative emotions such as anxiety, stress, or worries (Ang, Lau, et al., 2022; Gordon et al., 2022; Mavaddat et al., 2017; Smith et al., 2008; Steinhardt & Dolbier, 2008; Tamarit et al., 2022).

A meta‐analysis by Joyce et al. (2018) including 11 randomized‐controlled trials demonstrated that training programs incorporating elements from cognitive behavioral therapy and mindfulness‐based interventions can have promising effects on increasing an individual's level of resilience. While cognitive behavioral therapy focuses on changing maladaptive thinking patterns through strategies like cognitive reframing/reappraisal, emotional regulation, or psychoeducation (Kazantzis et al., 2018), mindfulness interventions are based on acceptance, relaxation, and the attempt to stay willfully present in the moment (O'Connor et al., 2023). Focusing on these aspects could increase resilience and help to find effective solutions for problems, as well as consider alternative perspectives during challenging times (Ang, Chew, et al., 2022).

Although resilience training programs were shown to be successful, it is still debated whether on‐site (face‐to‐face) interventions are more effective than online interventions in improving resilience (Ang, Chew, et al., 2022). However, on‐site intervention programs (e.g. Rich et al., 2022; Steinhardt & Dolbier, 2008) are difficult to attend for working individuals, students, and/or those with childcare responsibilities and were not feasible during the Covid‐19 pandemic. Especially during this time, resilience‐enhancing training programs were of great importance, as resilience has been shown to reduce the risk of depression and anxiety during the Covid‐19 pandemic and therefore mitigates the global mental health burden caused by outbreaks of infectious diseases (Yap et al., 2023). Thus, a self‐paced online resilience training provides the opportunity for training that is both time‐ and location‐independent, as well as easily accessible (e.g. by using a smartphone, computer, or tablet). Additionally, studies reported high effectiveness rates for online trainings, especially when the schedule of the program was flexible (Ang, Chew, et al., 2022).

### The present study

The focus of the present study lies primarily in promoting resilience, especially among working‐age individuals and students. As the living conditions of these adults are typically different from older ones (e.g. still working, childcare responsibilities, active social life, psychological stress caused by increased expectations induced by social media), and overall resilience was generally shown to be lower in younger adults (Gooding et al., 2012); studies on enhancing resilience in this specific group are of high importance.

Therefore, and to address the personal challenges faced by young adults, such as childcare responsibilities and a demanding work schedule, we provided a 6‐week online and flexible resilience training program comprising a set of 12 videos to strengthen the components of the seven pillars of resilience described above. The videos were psychoeducational and served as incentives to incorporate the presented ideas and strategies into everyday life. The focus is on strengthening participants' cognitive reframing skills to enhance self‐regulation, but also to help them build a personal toolkit of skills for coping with various daily life challenges. We hypothesized that participating in a 6‐week online resilience intervention would increase participants' resilience and reduce perceived stress and worries. We further investigated the relationship between training engagement and training satisfaction with potential (pre‐post) changes in resilience, perceived stress, and worries.

## METHOD

### Participants and recruitment

Participants were recruited via social media announcements and emails between February and April 2022. As the focus of the study was to improve resilience and alleviate negative symptoms such as stress and worries in working‐age individuals, an age range of 18 to 55 years was established as an inclusion criterion to recruit as many working or studying individuals as possible. The maximum age of 55 years was chosen because many individuals reduce their working hours before reaching the legal retirement age in Austria (65 for men and 60 for women). By doing so, we aimed to keep the focus of the study on working‐age individuals. In addition, only participants without a diagnosed psychological condition (e.g. depression or generalized anxiety disorder) were considered for participation.

The study was approved by the ethics committee of the University of Graz, Austria, (GZ. 39/26/63 ex 2021/22) and was conducted in accordance with the principles outlined in the Declaration of Helsinki, and all participants provided informed consent to take part in the study. They retained the right to withdraw from the study without providing any reason, and participants did not receive any compensation for taking part in the project.

**FIGURE 1:**
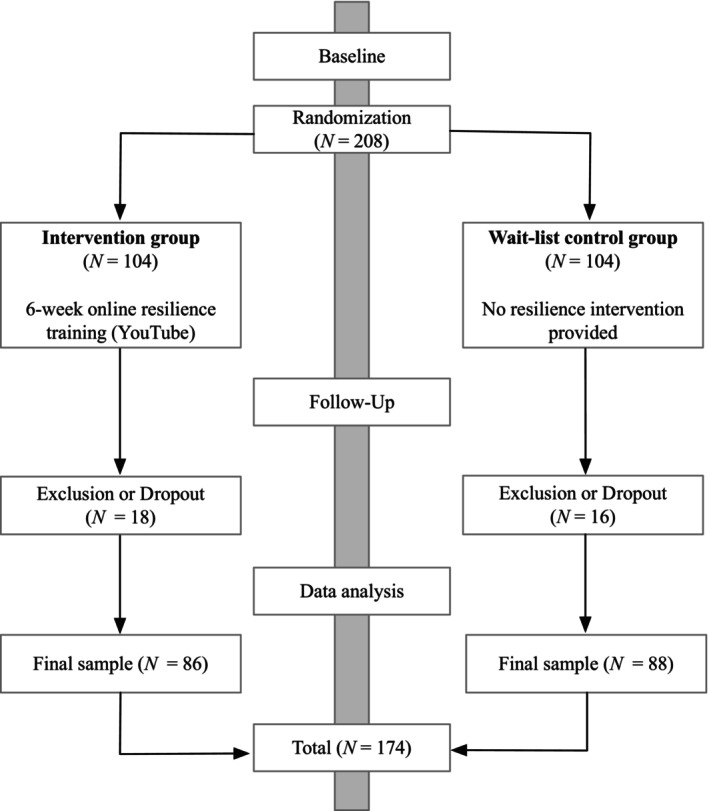
*Recruitment process. Note*. All analyses were conducted exclusively with the final sample (i.e. individuals who provided data at both time points).

The initial study sample comprised 208 participants (73.6% female) who were randomly and alternately assigned to either an intervention (*n* = 104) or a wait‐list control group (*n* = 104) by the study authors. As shown in Figure 1, 18 individuals from the intervention group, and 16 participants in the wait‐list control group were excluded (dropouts) as they ceased participation before completing the follow‐up survey. Those cases (missing data) were not included in our analyses. As a result, the final study sample was composed of 174 individuals, with 86 participants in the intervention group and 88 participants in the wait‐list control group, as illustrated in Figure 1. Consequently, the participation rate of the following study was 83.7%. Participants' ages ranged from 18 to 54 years (*M* = 24.05, *SD* = 5.29). The majority were female (74.1%) and currently university students (94.8%). Detailed demographic information for both groups is presented in Table 1.

**TABLE 1:** Demographic characteristics and major life events of study participants.

Variable	Intervention group	Control group	Significance
Age, mean (*SD*)	24.83 (5.93)	23.30 (4.49)	*p* = .057
Sex, *n* (% female)	61 (70.9)	68 (77.3)	*p* = .339
Education^a^, *n* (%)			*p* = .063
Apprenticeship	1 (1.2)	0 (0.0)	
High school diploma	48 (55.8)	65 (73.9)	
Bachelor's degree	27 (31.4)	18 (20.5)	
Master's degree	10 (11.6)	4 (4.5)	
PhD	0 (0.0)	1 (1.1)	
Student, *n* (% yes)	80 (93.0)	85 (96.6)	*p* = .327
Major life events, *n* (% yes)	29 (33.7)	31 (35.2)	*p* = .834

^a^ Highest education obtained so far. Values represent means and standard deviations (age) or frequencies, including corresponding percentages (sex, education, students, and life events). An independent samples *t*‐test was used to compare the two groups in terms of their age. Chi‐square tests were conducted to compare the other variables (sex, education, number of students, and major life events) between groups. No statistically significant differences were found between the groups, as *p*‐values exceeded values of .05 in all analyses.

### Procedure

**FIGURE 2:**
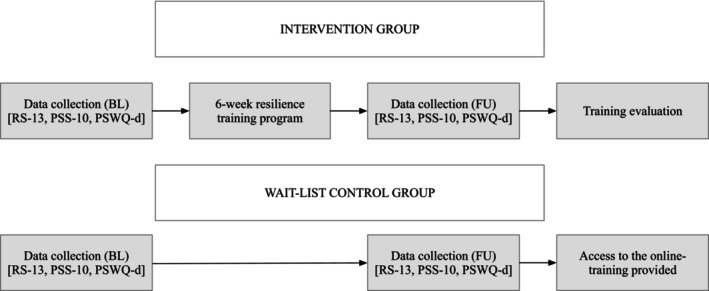
Study procedure.

Due to Covid‐19 restrictions, the resilience intervention was conducted online to adhere to the prevailing pandemic containment measures at that time. All participants were informed about the duration and purpose of the study as well as about their random allocation to one of the two groups (control group/intervention group). Both groups completed an online survey at two time points (before the study started: baseline; after 6 weeks: follow‐up) to gather demographic details and information on their level of resilience, perceived stress, and worries. Each survey required roughly 10 min to be completed. Individuals randomly assigned to the intervention group received a nonpublicly accessible YouTube link that contained a resilience training playlist consisting of 12 videos, each lasting about five to 10 min (average duration: 7 min). These videos were based on the seven pillars of resilience (Rampe, 2010) and developed by three psychologists from the Medical University of Graz, Austria (ML, DP, and VF) and reviewed by two experienced researchers with longstanding clinical expertise from the Medical University and the University of Graz. The video content was collaboratively developed to specifically target and strengthen the individual pillars of resilience. Additionally, the scripts for all videos were jointly reviewed, ensuring that high‐quality videos focusing on the individual pillars of resilience were created (Table 2). The videos aimed to enhance resilience and support dealing with stress, worries, and challenging situations. We recommended watching two videos per week and integrating the exercises and strategies into the participant's daily routine. In addition, we sent two reminder emails to the intervention group after 1 and 3 weeks to promote motivation and remind them to carry out the exercises. In the follow‐up survey, participants assigned to the intervention group were invited to evaluate the training program and each of the 12 videos. Participants randomly assigned to the wait‐list control group had no access to the resilience training playlist. However, after completing the follow‐up survey 6 weeks later, access to the resilience training program was provided by sending out the link via email. The entire study procedure is depicted in Figure 2.

**TABLE 2:** Short description of each video provided in the resilience training program.

Week	Video (English title)	Description	Pillar of resilience (Rampe, 2010)
1	Energy management	The focus of this exercise was on optimizing the use and control of resources, particularly personal energy	Leaving the victim role, taking responsibility, and planning for the future
	Setting SMART goals	We tried to encourage individuals to create objectives that are specific (S), measurable (M), achievable (A), relevant (R), and time‐bound (T)	Solution orientation and planning for the future
2	The ABC (DE) model	Individuals learned about the cognitive behavioral therapy (CBT) approach developed by Albert Ellis. A: Activating event, B: Beliefs, C: Consequences	Solution orientation, leaving the victim role, and taking responsibility
	Hunting the good stuff	The focus was on seeking (“hunting”) for positive experiences in life (e.g. in daily routines, on the way to work, or at home) and finding joy in the little things. The positive experiences could be collected, for instance, in a jar	Optimism
3	Mastering challenges creatively	The task of this exercise was to generate as many creative and positive reappraisals for problems or challenges as possible	Optimism, leaving the victim role, and taking responsibility
	Mindfulness meditation	A relaxation exercise/mindfulness meditation was provided	Acceptance
4	Strength through social relationships	In this task, the focus was on considering which individuals (friends, acquaintances, and family) primarily contribute energy or deplete energy, with the aim of strengthening positive connections	Solution orientation, leaving the victim role, and network orientation
	Stopping worries	This exercise targeted resilient thinking. Participants were asked to evaluate whether their thinking patterns are helpful, constructive, or predominantly detrimental to themselves	Acceptance and leaving the victim role
5	Success journal	Participants were encouraged to contemplate the meaning of success for themselves. Over the course of several days, they were instructed to record 2–3 successes in writing, for instance, in a notebook	Optimism and leaving the victim role
	Language and well‐being	In this task, we emphasized how important language, especially self‐talk, is for our well‐being	Acceptance, leaving the victim role, and taking responsibility
6	Autogenic training	A relaxation exercise/autogenic training was provided	Acceptance
	Acceptance	The final task addressed the theme of “acceptance.” It involved accepting things that we cannot change and finding solutions for situations over which we have control	Acceptance and leaving the victim role

### Resilience intervention

The resilience training program was based on the theoretical framework of Rampe's (2010) seven pillars of resilience. This framework was chosen because it offers a comprehensive overview of resilience, considering it from multiple perspectives, with various exercises that can be developed for each pillar. In addition, the pillars of resilience summarize different factors of highly resilient individuals, which are similar to those proposed by other researchers (Joyce et al., 2018; MacLeod et al., 2016). As proposed by a previous meta‐analysis (Joyce et al., 2018), the intervention comprised a mix of exercises from cognitive behavioral therapy (e.g. reframing, detecting maladaptive thought patterns, and Ellis' ABCDE thinking model) and mindfulness training programs (e.g. meditation and relaxation exercises). Therefore, the training program incorporated key elements of resilience and offered strategies to change the perspective on challenging situations (reframing, ABC model, and stopping rumination), techniques to strengthen optimism and acceptance (journaling positive moments and achievements and accepting unchangeable situations), acquiring energy management, relaxation techniques (autogenic relaxation), and a set of other exercises (Table 2). Participants had the flexibility to watch the videos at any time and as often as they desired. In line with previous studies on this topic, the training duration was set to 6 weeks, stating that a training period of 4–8 weeks is sufficient to achieve positive results (Golding et al., 2015; Lin et al., 2019; Lo et al., 2018; Mavaddat et al., 2017; Sadler et al., 2017; Steinhardt & Dolbier, 2008).

### Assessment

#### Resilience

The level of resilience was assessed by using the German version of the 13‐item resilience scale (RS‐13; Leppert et al., 2008). Each item of the RS‐13 is rated on a 7‐point Likert scale ranging from 1 (*I do not agree*) to *7* (*I totally agree*), resulting in an overall score ranging from 13 to 91. Higher scores indicate a higher level of psychological resilience. An overall score of 73–91 is considered high, 67–72 as moderate, and values of 66 or below indicate a low level of resilience. Reliability indices for the scale have generally been shown to be good (Leppert et al., 2008), including those found in this study for both pre‐assessment (ω = .809, α = .816) and post‐assessment (ω = .853, α = .857). Furthermore, this questionnaire has been used in similar populations, such as undergraduate students (McLafferty et al., 2012) and was shown to be a reliable and valid instrument (Rosendahl et al., 2024).

#### Perceived stress

The German version of the Perceived Stress Scale (PSS‐10; Schneider et al., 2020) was utilized to assess participants' levels of stress by using a 5‐point Likert scale ranging from 1 (*never*) to 5 (*very often*). The PSS‐10 comprises two subscales (perceived self‐efficacy and perceived helplessness) to evaluate how participants comprehend situations as uncontrollable as well as their subjective coping abilities (Schneider et al., 2020). Both subscales as well as the global score (ranging from 10 to 50) were used as an outcome in this study. Higher scores reflect higher levels of perceived stress. Reliability coefficients were calculated for each subscale separately and were good for both the perceived helplessness subscale (ω_pre_ = .825, ω_post_ = .854; α_pre_ = .825, α_post_ = .853) and the perceived self‐efficacy subscale (ω_pre_ = .751, ω_post_ = .771; α_pre_ = .704, α_post_ = .759). Additionally, a review article highlighted that the PSS is an easy‐to‐use questionnaire with acceptable psychometric properties (Lee, 2012) and has demonstrated adequate reliability and validity in different populations (Bastianon et al., 2020; Koğar & Koğar, 2023; Maroufizadeh et al., 2018; Soria‐Reyes et al., 2022).

#### Worries

Worries were evaluated by using the German version of the Penn State Worry Questionnaire (PSWQ‐d; Glöckner‐Rist & Rist, 2006). Participants were asked to rate 16 questions like “I worry all the time” or “My worries overwhelm me” on a Likert scale ranging from 1 (*not at all typical of me*) to 5 (*very typical of me*). The summation of item scores yields an overall score ranging between 16 and 80, with higher values indicating heightened levels of rumination and worrying. Studies have shown that the questionnaire exhibits high reliability and validity (Meyer et al., 1990; Stöber, 1998) and has also been used in various populations, such as a clinical anxiety disorders sample (Brown et al., 1992). Also in the present study, excellent reliability coefficients were found for both the pre‐assessment (ω = .930, α = .924) and the post‐assessment (ω = .924, α = .922).

#### Major life events

To control for the potential impact of significant life events on resilience, perceived stress, and worry, participants were asked to indicate whether any positive (e.g. entering a new relationship and getting married) or negative (e.g. experiencing a divorce and the death of a close relative) life event occurred during the study period. This was assessed using a binary yes or no question. Therefore, we were able to ensure comparability between both groups.

#### Training evaluation

Upon conclusion of the study, participants randomly assigned to the intervention group had the option to rate (1) if they adhered to the suggested order in which the YouTube videos should be viewed (yes/no), (2) the extent to which they engaged with the training program on an 11‐point Likert scale (0 = *not at all* to 10 = *very intensively*), (3) their overall satisfaction with the intervention (0 = *not at all* to 10 = *very satisfying*), and (4) their evaluation of each individual exercise on a 4‐point Likert scale, ranging from 0 (*not helpful*) to 3 (*very helpful*).

### Sample size calculation

The required sample size was calculated using the G*Power software (Version 3.1; Faul et al., 2007). In order to achieve a statistically significant interaction effect (ANCOVA; group*time, which is the primary effect of interest), a minimum sample size of 128 individuals is required (ɑ = 0.05, Power [1‐β] = 0.80, *f* = .25). A moderate predicted effect size for resilience interventions was chosen based on the result of a previous meta‐analysis (Joyce et al., 2018). This implies a sample of at least 64 individuals assigned to each group to detect a significant group * time interaction effect of medium size.

### Statistical analysis

We performed complete‐case analyses using IBM SPSS Statistics (Version 28). The study was based on a 2 × 2 mixed‐factorial design with group assignment (intervention group vs. wait‐list control group) as the between‐subject factor and measurement time point (baseline vs. follow‐up) as the within‐subject factor. Additionally, we controlled for age, sex, and life events within our analyses to obtain robust results. Covariates were centered before entering them into the analysis (Schneider et al., 2015). We therefore performed univariate analyses of covariance (ANCOVA) for each outcome variable (resilience, perceived stress, and worries). Additionally, a multivariate analysis of covariance (MANCOVA) with univariate ANCOVAs as post hoc tests was performed for the perceived stress subscales (perceived self‐efficacy and perceived helplessness), as both scales ought to measure the same global construct. The assumption of homogeneous variances at both time points was assessed using Levene's test, and the assumption of homogeneity of variance–covariance matrices was examined through the Box's M test. Assumptions for ANCOVA (i.e. homogeneity of regression slopes and no group differences on the covariate) were assessed before conducting the analyses. To counteract an alpha error inflation due to multiple testing, Bonferroni correction was applied for all pairwise comparisons. Furthermore, we computed Pearson correlations to investigate associations between training engagement/satisfaction and pre‐post changes in resilience, perceived stress, and worries. Finally, correlations between the change scores of our outcomes (resilience, perceived stress, and worries) were calculated.

## RESULTS

Both the intervention and wait‐list control group did not show any significant differences in age (*t*[158.24] = −1.92, *p* = .057, *d* = −0.29), sex (χ^2^[1] = 0.91, *p* = .339, φ_c_ = .07), education (χ^2^[4] = 8.91, *p* = .063, φ_c_ = .06) or the number of university students (χ^2^[1] = 1.13, *p* = .327, φ_c_ = .08). They were also comparable regarding the number of positive or negative life events (intervention group: 29 events, control group: 31 events) experienced during study participation (χ^2^[1] = 0.04, *p* = .834, φ_c_ = .02). Descriptive statistics for both groups at baseline and follow‐up are presented in Table 3. With regard to the participants' training engagement, approximately 1.300 clicks were recorded in the YouTube analytics throughout the training period. This means that, on average, each participant generated around 15 clicks (1300 clicks/86 participants), suggesting that they engaged with multiple videos or watched some of them more than once. However, data protection regulations did not allow tracking how often each individual participant clicked on a specific video.

**TABLE 3:** Descriptive statistics for resilience, perceived stress, and worries.

Variable	Intervention group (*n* = 86)				Wait‐list control group (*n* = 88)			
	Baseline		Follow‐up		Baseline		Follow‐up	
	M	SD	M	SD	M	SD	M	SD
Resilience	66.17	9.95	69.85	9.88	66.99	9.30	66.16	9.64
Perceived stress	29.22	6.31	25.13	6.28	27.83	5.69	28.40	6.33
Helplessness	18.85	4.15	16.13	4.50	17.61	4.18	17.76	4.54
Self‐efficacy	13.63	2.64	15.00	2.33	13.78	2.02	13.36	2.36
Worries	51.52	12.08	46.52	11.08	51.63	11.64	50.60	11.82

*Note*: Values represent means (*M*) and standard deviations (*SD*), separately for the intervention and the wait‐list control group.

### Resilience

**FIGURE 3:**
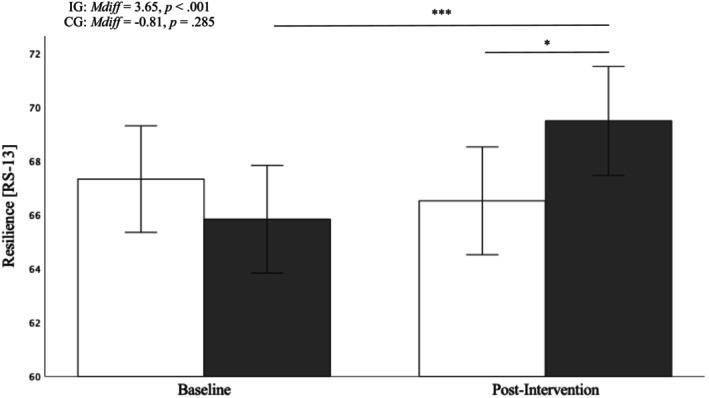
*Group differences in mean resilience scores at the baseline and follow‐up assessment. Note*. White bars represent the control group (CG), black bars represent the intervention group (IG). Error bars represent 95% confidence intervals (CI). * *p* < .05, ** *p* < .01, *** *p* < .001.

Our analysis yielded a significant main effect for time, a nonsignificant main effect for group, and a significant group * time interaction (Table 4). Bonferroni‐adjusted post hoc analyses revealed that both groups did not differ in their adjusted mean resilience scores at baseline (*p* = .301) but did significantly differ after 6 weeks (*M*_*diff*_ = 2.97, *p* = .042), indicating higher resilience scores in the intervention group postintervention compared to the wait‐list control group. The latter showed no increase in resilience from baseline to follow‐up (*p* = .285), whereas the mean resilience score of the intervention group did increase significantly between the measurement time points (*M*_*diff*_ = 3.65, *p* < .001), illustrated in Figure 3.

**TABLE 4:** ANCOVA results for training effectiveness on resilience, stress, and worries.

Domain	Effect	*F*	df	Error df	*p*	η_p_^2^
**Resilience**	Time	7.19	1	169	.008	.041
	Group	0.31	1	169	.581	.002
	Time × group	17.21	1	169	<.001	.092
**Perceived stress** **(global score)**	Time	17.77	1	169	<.001	.080
	Group	0.46	1	169	.498	.003
	Time × group	27.10	1	169	<.001	.138
**Perceived self‐efficacy**	Time	6.56	1	169	.011	.037
	Group	3.89	1	169	.050	.023
	Time × group	24.23	1	169	<.001	.125
**Perceived helplessness**	Time	16.33	1	169	<.001	.088
	Group	0.01	1	169	.928	<.001
	Time × group	21.39	1	169	<.001	.112
**Worries**	Time	32.17	1	169	<.001	.160
	Group	0.40	1	169	.530	<.001
	Time × group	14.99	1	169	<.001	.081

*Note*: small effect: η_p_^2^ = 0.01, medium effect: η_p_^2^ = 0.06, large effect: η_p_^2^ = 0.14.

### Perceived stress

**FIGURE 4:**
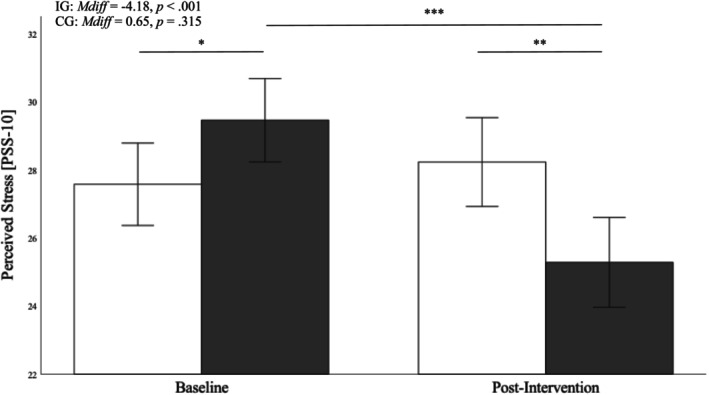
*Group differences in mean perceived stress scores at the baseline and follow‐up assessment. Note*. White bars represent the control group (CG), black bars represent the intervention group (IG). Error bars represent 95% confidence intervals (CI). * *p* < .05, ** *p* < .01, *** *p* < .001.

We found a significant main effect for time, a nonsignificant main effect for group, and a significant group * time interaction (Table 4). Both groups did differ in their adjusted average perceived stress scores at baseline (*p* = .033) and after 6 weeks (*M*_*diff*_ = 2.95, *p* = .002). On average, participants assigned to the intervention group had significantly lower perceived stress scores postintervention than those in the wait‐list control group. Importantly, pairwise comparisons confirmed that the adjusted perceived stress scores in the wait‐list control group did not significantly change over time (*p* = .315), whereas a significant decrease in stress was observable in the intervention group (*M*_*diff*_ = 4.18, *p* < .001). The results are visualized in Figure 4.

To provide further insight into changes within participants' perceived stress levels, both PSS‐10 subscales (*perceived self‐efficacy* and *perceived helplessness*) were examined separately. Both subscales were highly correlated to the same amount at both time points (baseline: *r* = −.67; follow‐up: *r* = −.67). The multivariate test statistics (Pillai's Trace) indicated a significant main effect for time (*V* = .09, *F*[2, 168] = 8.12, *p* < .001, η_p_^2^ = .088), a significant main effect for group (*V* = .04, *F*[2, 168] = 3.52, *p* = .032, η_p_^2^ = .040), and a significant group * time interaction effect (*V* = .14, *F*[2, 168] = 13.97, *p* < .001, η_p_^2^ = .143). Univariate follow‐up analyses revealed significant interaction effects for both the perceived self‐efficacy and perceived helplessness subscales (Table 4). Both groups did not differ on the self‐efficacy subscale at baseline (*p* = .326) but differed on the helplessness subscale (*p* = .013). Additionally, we found significant between‐group differences at follow‐up (self‐efficacy: *p* < .001; helplessness: *p* = .036). Only the intervention group was able to decrease their perceived helplessness (*M*_*diff*_ *=* −2.78, *p* < .001) and increase their level of perceived self‐efficacy (*M*_*diff*_ *=* 1.40, *p* < .001), while the adjusted subscale values remained stable in the control group (*p* = .652, *p* = .089).

### Worries

**FIGURE 5:**
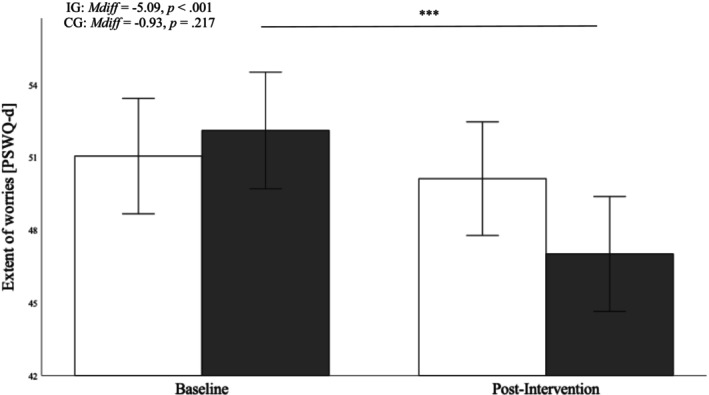
*Group differences in the extent of worries before and after the intervention. Note*. White bars represent the control group (CG), black bars represent the intervention group (IG). Error bars represent 95% confidence intervals (CI). * *p* < .05, ** *p* < .01, *** *p* < .001.

Again, the analysis yielded a significant main effect for time, a nonsignificant main effect for group, and a significant group * time interaction (Table 4). Bonferroni‐adjusted post hoc analyses indicated that there were no adjusted group differences at baseline (*p* = .540) and at the follow‐up time point (*M*_*diff*_ = 3.10, *p* = .069). However, whereas the wait‐list control group was, on average, not able to decrease their worries (*p* = .217), the intervention group reduced their adjusted average amount of worries by 5 points on the utilized questionnaire (*M*_*diff*_ = 5.09, *p* < .001), as demonstrated in Figure 5.

### Association between resilience, perceived stress, and worries

There were significant correlations between all outcome measure change scores. First, an increase in resilience from baseline to follow‐up was associated with a decrease in perceived stress (*r* = −.42, *p* < .001) and a decline in the extent to which participants worried (*r* = −.41, *p* < .001). In addition, a decrease in the amount of perceived stress between both time points was further associated with a reduction in participants' worries (*r* = .45, *p* < .001).

### Training evaluation and exploratory analyses

Upon completion of the follow‐up survey, we asked participants assigned to the intervention group to evaluate the training as well as the individual exercises. 88.2% (*n* = 85) reported adhering to the recommended training schedule of watching two videos per week. Assessed on an 11‐point Likert scale (0–10, higher scores indicating higher training engagement or higher training satisfaction), the mean training engagement was 5.96 (*SD* = 1.77, *Mdn* = 6.00), and the training was found to be satisfactory (*M* = 7.05, *SD* = 2.15, *Mdn* = 7.00). The two best rated exercises included as follows:

- “Hunting The Good Stuff”: an exercise that entails “hunting” for positive experiences during the day and recording them in a journal or a glass to create a collection of enjoyable experiences.
- “Mastering Challenges Creatively”: an exercise related to cognitive reappraisal strategies, requiring participants to identify innovative, positive, and optimistic alternatives to a specific problem or situation.

Finally, training engagement was neither associated with changes in resilience (*r* = .14, *p* = .228), perceived stress (*r* = .03, *p* = .784), nor changes in worries (*r* = −.06, *p* = .595). In addition, an individual's satisfaction with the training was not associated with changes in resilience scores (*r* = .06, *p* = .560), perceived stress (*r* = .10, *p* = .392), or worries (*r* = .03, *p* = .821). However, a clear correlation between training engagement and training satisfaction was present (*r* = .57, *p* < .001), suggesting that those more satisfied with the training showed more effort to engage in the exercises and implement them into their daily routines or vice versa.

## DISCUSSION

Interventions to strengthen psychological resilience have great potential to improve well‐being. Previous research highlighted the importance of online resilience interventions (e‐health interventions; Joyce et al., 2018; Ang, Chew, et al., 2022), which circumvent the logistic challenges associated with on‐site resilience training programs (e.g. coordination of groups, fixed training times, and rent for training facilities). Consequently, this study examined the effectiveness of a 6‐week online resilience training program in working‐age (18–55 years) individuals during the global Covid‐19 pandemic, which led to various mental health‐related issues such as increased stress (Droit‐Volet et al., 2020; Lakhan et al., 2020), depression, or anxiety (Lakhan et al., 2020). In addition, working‐age individuals face a multitude of challenges, such as childcare responsibilities and work demands, making studies promoting positive thinking patterns and behaviors in a flexible, asynchronous online setting highly important.

We found that participating in the intervention group was associated with higher levels of resilience and decreased levels of perceived stress and worries postintervention when compared to the wait‐list control group. Our participants, consisting mostly of university‐aged women, could enhance their resilience by approximately 3.65 points and reduce negative emotions such as perceived stress and worries by 4.18 and 5.09 points, respectively. The magnitude of those changes was comparable to those observed in other studies (e.g. Deckro et al., 2002; Luo et al., 2021) and was in line with meta‐analyses, indicating a small to moderate effect of resilience intervention programs (Ang, Lau, et al., 2022; Joyce et al., 2018). Thus, the positive outcomes and effect sizes validate the effectiveness of our online resilience intervention. We could further replicate the common finding that resilience is negatively correlated with stress (Connor & Davidson, 2003; Gyawali et al., 2020; Steinhardt & Dolbier, 2008) and worries (Gordon et al., 2022; Tamarit et al., 2022), suggesting that highly resilient individuals might perceive less stress and negative thoughts.

Our training program included 12 short videos with ideas and incentives that could be integrated into everyday life. Various components were strengthened, such as cognitive flexibility (i.e. considering alternative perspectives), self‐efficacy (believing in one's abilities), problem‐solving (generating promising solutions to overcome problems and challenges), and social skills (forging and strengthening meaningful relationships) (Ang, Chew, et al., 2022). Advantages of the online training are the ability to reach a large number of individuals, easy access to the training (compared to in‐person sessions), high flexibility, and reduced time constraints for participants through providing an asynchronous training program. Studies with such flexible program schedules were found to be superior compared to those with fixed schedules (Ang, Chew, et al., 2022). This enabled participants to complete the training at their own preferred pace and to view the videos anytime and from any location.

In the digital age, easily accessible and widely used online platforms, such as YouTube, are well suited for offering such training opportunities, as they make it possible to reach a large portion of the population. One disadvantage of online training programs, however, lies in the accessibility of such programs for older individuals, as internet access may pose a challenge for them. However, there were no difficulties for the sample participating in this study. Finally, it should be noted that online self‐paced programs rely on the motivation of participants (Hartnett, 2016). Hence, future studies might consider motivational incentives and ideas. Nevertheless, we assume our intervention was effective, as it offered a broad range of skills that have been successfully applied in routine clinical practice. Participants were encouraged to individually try out a specific strategy (e.g. hunting the good stuff or success journal) for a defined period (e.g. 1 week). This approach increased the likelihood of continuing strategies they found most helpful for themselves.

Compliance with our training was high, with nearly 9 out of 10 participants reporting adherence to the recommended training order, and YouTube analytics revealed roughly 1.300 clicks generated during the training period. In addition, training engagement (on average 5.96/10 points) and training satisfaction (on average 7.05/10 points) were moderate to high, as rated by the intervention group upon completing the study. Astonishingly, neither training engagement nor an individual's satisfaction with the training program was significantly associated with training successes (i.e. an increase in resilience or a decrease in perceived stress and worries). We concluded that participants might especially benefit from incentives and ideas provided in the exercises (e.g. adopting a different perspective and acquiring reframing strategies), regardless of the amount of time they spent on the exercises or how satisfying the training was perceived to be. Such incentives can be easily provided through short and informative online videos.

Although training time is generally considered a crucial factor during new skill acquisitions (Joyce et al., 2018), including learning new strategies as provided in resilience interventions, incentives, and suggestions of specific exercises might have led to the present effects. This could be a particular advantage for working‐age participants, as even very short incentive videos are sufficient to integrate resilience‐promoting strategies and behaviors into everyday life. Furthermore, we provided sufficient time to integrate the training content into the daily routine, as the training duration was set to 6 weeks, comparable to the period (4–8 weeks) of other research studies (Golding et al., 2015; Lin et al., 2019; Lo et al., 2018; Mavaddat et al., 2017; Steinhardt & Dolbier, 2008).

One strength of this study is its large sample size with a total of 174 participants. This is noteworthy, as approximately 50% of the digital resilience promotion studies conducted so far had fewer than 50 participants per group (control group and intervention group) (Ang, Chew, et al., 2022), which eventually limits the generalizability of the results published so far. Although attrition rates were found to be a major problem in online mental health interventions (Litvin et al., 2020), the attrition rate in our study was small, namely, 16.3%. Another asset to highlight is the asynchronous training design (Ang, Chew, et al., 2022), as participants had the opportunity to watch the videos at any time and independent of their current location. The provision of location‐independent interventions, such as online training programs, is also beneficial in overcoming logistic challenges (e.g. training times, arrival to the training location, and rents for training locations). This opportunity provides a simple and convenient way to implement new habits and strategies as conveyed in our training program. Additionally, the study's randomized allocation to either an intervention or wait‐list control group ensured comparability between the two groups with respect to demographic characteristics. However, the study's generalizability is limited by the fact that most participants were female (74.1%) and university students (94.8%), suggesting that young women may be more likely to engage in resilience‐enhancing activities. Furthermore, a small number of participants (around 5%) were not students, which may have influenced our findings, as students might experience different stressors and resilience‐related challenges compared to nonstudents. Future studies could aim to include more non‐student participants to improve the external validity of the findings. Additionally, as recruitment was carried out via social media announcements and emails, it is possible that older individuals without access to the internet (e.g. through computers or smartphones) were excluded from potential training programs. Future studies could specifically test the effectiveness of such programs in an older population. A general limitation of online interventions (as opposed to individual face‐to‐face training) is that we were unable to monitor how often each participant watched the videos or whether the exercises were implemented as we intended. However, the positive results of the study suggest that this was the case.

In addition, it remains uncertain whether a mean change in resilience scores in a questionnaire is truly indicative of the ability to bounce back from challenging or demanding events. Chmitorz et al. (2018) suggested that resilience may only be reliably assessed in individuals currently exposed to significant trauma or stress, highlighting the need for resilience interventions in specific high‐risk groups such as military or police personnel, or people experiencing adverse life events, such as a stroke (Gyawali et al., 2020). These specific groups have a high risk of experiencing a stressful or demanding situation and might particularly benefit from resilience interventions. Finally, we encourage future studies to extend our research questions with implications for mental health in general.

### Implications

Although previous research established a framework for future resilience intervention studies (e.g. Chmitorz et al., 2018), little is known about a general theoretical guideline regarding the development of training programs. This led to the use of different theoretical approaches in various intervention studies (Leppin et al., 2014). Currently, there is no general agreement on how long a training program should be, which exercises are most effective, or in which context they should take place (e.g. e‐health interventions vs. face‐to‐face, individual vs. group sessions). It is likely, however, that the most effective presentation mode differs from person to person. Furthermore, given the broad construct of resilience along with individual strengths and weaknesses, a variety of exercises combining cognitive behavioral therapy and mindfulness training seem most promising (Joyce et al., 2018). The results of our study show that exercises from cognitive behavioral therapy (e.g. acquiring reappraisal strategies) and shifting the focus (e.g. keeping a journal diary) were rated as the most effective exercises in our cohort. Although these exercises were developed based on Rampe's (2010) seven pillars of resilience model, it is important to note that the pillars are just one of many options to structure the multiple strategies linked to resilience. Nevertheless, they also overlap with the core elements of the American Psychological Association's 10 steps to resilience and the national guidelines to foster resilience. In the future, feedback from participants (e.g. which exercises were most effective) and factors influencing successful strategies (e.g. age, gender, occupation) will help to develop more targeted interventions. Additionally, exploring which distinct components are most effective in specific cohorts will enhance our understanding of the complex process of resilience. Finally, as stated above, the assessment and training of resilience in high‐risk groups (e.g. police, military, and clinical populations) is important (Chmitorz et al., 2018), as these groups are often exposed to intense stressors that may lead to significant mental health issues. Since resilience refers to the ability to adapt and recover in the face of adversity, enhancing resilience in these high‐risk populations is crucial and may help to effectively cope with and recover from the unique stressors they face in their daily lives.

## CONCLUSIONS

The quote, “Happiness is not the absence of problems; it is the ability to deal with them” (Steve Maraboli) illustrates the key attribute of highly resilient individuals. In this study, we evaluated the effectiveness of a 6‐week online resilience intervention on a variety of psychological outcomes. Our findings indicate that participating in a resilience training program can significantly improve psychological resilience and is accompanied by a reduction in perceived stress and worries. This highlights that resilience is malleable and can be strengthened through training. Therefore, flexible online training programs designed to enhance resilience provide a valuable opportunity to acquire new strategies and help to potentially manage ongoing challenges more effectively.

## AUTHOR CONTRIBUTIONS

**Manuel Leitner:** Conception and design; acquisition of data; analysis and interpretation of data; drafting the manuscript. **Andreas Fink:** Conception and design; interpretation of data; revising the manuscript critically for important intellectual content. **Viktoria Fruhwirth:** Conception and design; interpretation of data; revising the manuscript critically for important intellectual content. **Stefanie Hechenberger:** Interpretation of data; revising the manuscript critically for important intellectual content. **Christian Enzinger:** Interpretation of data; revising the manuscript critically for important intellectual content. **Daniela Pinter:** Conception and design; interpretation of data; revising the manuscript critically for important intellectual content.

## CONFLICT OF INTEREST STATEMENT

The authors report there are no competing interests to declare.

## ETHICS STATEMENT

The study was approved by the ethics committee of the University of Graz, Austria (GZ. 39/26/63 ex 2021/22) and was conducted in accordance with the principles outlined in the Declaration of Helsinki, and all participants provided informed consent to take part in the study. They retained the right to withdraw from the study without providing any reason, and participants did not receive any compensation for taking part in the project.

## Data Availability

The data that support the findings of this study are available from the corresponding author upon reasonable request.
